# Editorial

**Published:** 1876-05

**Authors:** 


### Editorial.

It will hardly be necessary to introduce the subject matter of the following letters with anything more than a statement of the facts connected with the manner of our possession of them.

Early in March, 1876, the janitor of the Chicago Medical College received a note from the Dean of the Kentucky School of Medicine, requesting him to send the names of a number of the students of the former institution to him.

The janitor complied, and being of an inquiring turn of mind wrote and mailed the following letter to the Dean of the Kentucky School of Medicine. We mark it

(No. 1.) CHICAGO, March 5, 1876.

*Dear Sir*—I have attended one year in the Chicago Medical College, and according to the rules of this institution I shall have to spend two more years before I can graduate. Now I want to get through in shorter time in your college; will you then oblige me by stating so, and send me your annual announcement. Respectfully yours,

P. NILSON, 785 Wabash Ave.

To this a letter, of which the following is an exact copy, came as an answer:

(No. 2.) LOUISVILLE, March 9, 1876.

MR. P. NILSON: *Dear Sir*—I am just in receipt of your letter of the 5th inst. As you have attended one course of lectures, you would be allowed to come forward for graduation in this school after attending one more course. You can come on here now and join the present clas and come forward in June of this year for graduation. I send you a catalogue.

Yours very truly, E. S. GAILLARD.

Letters Nos. 3 and 4 were handed to us by Prof. Nelson of the Chicago Medical College, who vouches for their genuineness.

(No. 3.) CHICAGO, March 21, 1876.

*To the Dean of the Louisville Medical College, Louisville, Ky.:*

Sir—I am desirous of attending college this summer. I address you in relation thereto.

I am a man of 25 years; my early education I obtained in Indiana and my classics in Rockford, Illinois. I began the study of medicine in December, 1874. I have attended a course of summer lectures of three months, and a winter course of six months, both courses being taken in Chicago Medical College.

I have creditably passed the Junior examinations and completed three dissections of the human body.

Now will you admit me as a student of your college and at the same time allow me to come up as a candidate for graduation, and graduate at the end of this present session; providing I pass the required examinations and furnish certificate of moral character.

As I have been detained for examination in the Chicago Medical College which closes this 21st inst., your immediate answer will oblige

Yours truly, M. M. ROWLEY.

P. S. Please send me catalogue to 45 South Clark Street, Chicago.

Letter No. 4 came in answer to the above.

LOUISVILLE, Ky., March 24, 1876.

MR. M. M. ROWLEY:

*Dear Sir*—Your letter of the 21st inst. has just been received. You can enter the present session of this college and come forward for graduation with the class in June; I would advise you, however, to come on with the least possible delay, as the course was begun on the first of this month. I send you a catalogue by this mail.

Yours very truly, E. S. GAILLARD.

Commentary upon the above letters is entirely unnecessary. They are plain and to the point.

